# Supplementary material for: AI/ML-based prediction of TB treatment failure: A systematic review and meta-analysis
Source: medRxiv. 2026 Apr 22:2026.04.16.26350453. Preprint. [Version 1] doi: 10.64898/2026.04.16.26350453 (PMC13131719; doi:10.64898/2026.04.16.26350453)
Supplement: Supplement 5 [file NIHPP2026.04.16.26350453v1-supplement-5.pdf]

medRxiv preprint doi: https://doi.org/10.64898/2026.04.16.26350453; this version posted April 22, 2026. The copyright holder for this preprint (which was not certified by peer review) is the author/funder, who has granted medRxiv a license to display the preprint in perpetuity.
It is made available under a CC-BY 4.0 International license .

Supplementary information:

Figure. PRISMA Flow Diagram
Flow diagram documenting the study selection process for the systematic review and meta-analysis. From an initial 1,672 records identified through database searching, 1,344 records were removed during initial filtering based on study focus (non-tuberculosis), language, non-article publication types, irrelevant categories, missing metadata, or retraction status. After removal of 11 duplicates, 317 abstracts were screened, of which 265 were excluded as not relevant (review studies, animal models, tuberculosis-focused but not prediction models). Fifty-two full-text articles were assessed for eligibility; 18 were excluded for using traditional statistical methods without a machine learning component or not focusing on treatment failure prediction. Thirty-four studies were included in the systematic review; 19 reported area under the curve values and were included in the meta-analysis.

Table S1: Study characteristics

Table S2: Probast study level

Table S3: Study characteristics, cont'd.

S4 File: PROSPERO Registered Protocol

S5 – Search strategy

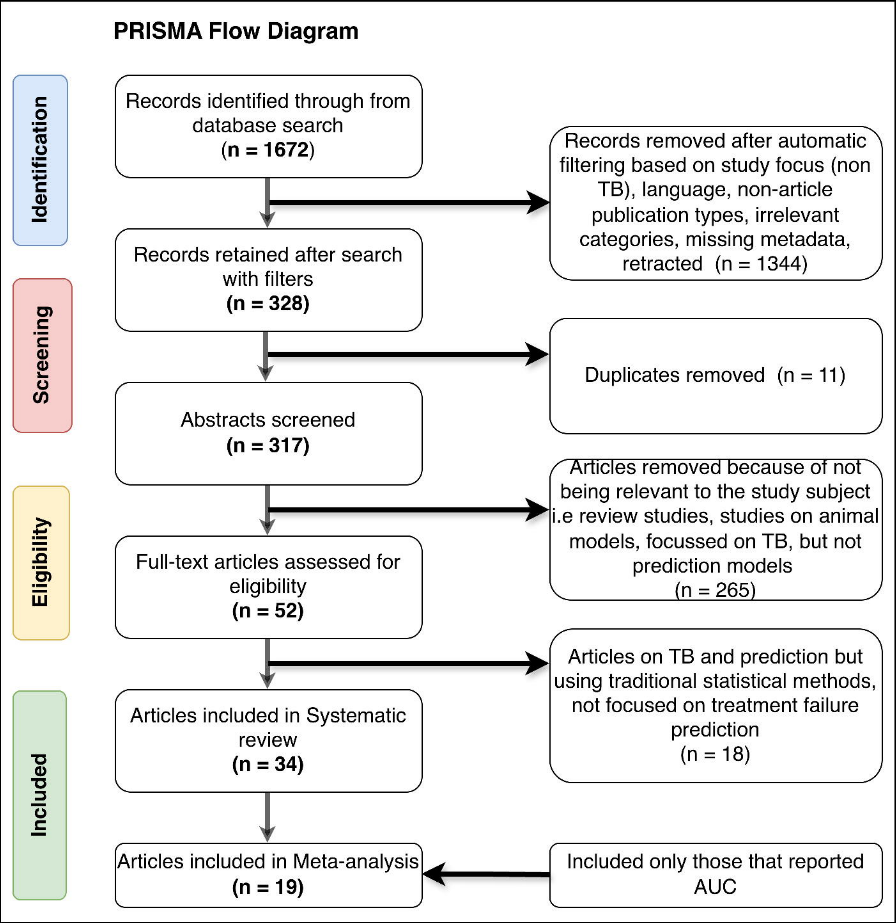
PRISMA Flow Diagram
Identification
Screening
Eligibility
Included
Records identified through from database search (n = 1672)
Records removed after automatic filtering based on study focus (non TB), language, non-article publication types, irrelevant categories, missing metadata, retracted (n = 1344)
Records retained after search with filters (n = 328)
Duplicates removed (n = 11)
Abstracts screened (n = 317)
Articles removed because of not being relevant to the study subject i.e review studies, studies on animal models, focussed on TB, but not prediction models (n = 265)
Full-text articles assessed for eligibility (n = 52)
Articles on TB and prediction but using traditional statistical methods, not focused on treatment failure prediction (n = 18)
Articles included in Systematic review (n = 34)
Articles included in Meta-analysis (n = 19)
Included only those that reported AUC
